# SGK1 affects RAN/RANBP1/RANGAP1 via SP1 to play a critical role in pre-miRNA nuclear export: a new route of epigenomic regulation

**DOI:** 10.1038/srep45361

**Published:** 2017-03-30

**Authors:** Vincenzo Dattilo, Lucia D’Antona, Cristina Talarico, Mjriam Capula, Giada Catalogna, Rodolfo Iuliano, Silvia Schenone, Sante Roperto, Cataldo Bianco, Nicola Perrotti, Rosario Amato

**Affiliations:** 1 University “Magna Graecia” of Catanzaro, Dept. of “Scienze della Salute”, Viale Europa Catanzaro, Italy; 2 University “Magna Graecia” of Catanzaro, Dept. of “Medicina Sperimentale e Clinica”, Viale Europa Catanzaro, Italy; 3 University of Genova, Dept of Farmacia, Viale Benedetto XV 3, Genova, Italy; 4 University “Federico II” of Naple, Dept of Medicina Veterinaria e Produzioni Animali, Via Federico Delpino 1, Napoli, Italy.

## Abstract

The serum- and glucocorticoid-regulated kinase (SGK1) controls cell transformation and tumor progression. SGK1 affects mitotic stability by regulating the expression of RANBP1/RAN. Here, we demonstrate that SGK1 fluctuations indirectly modify the maturation of pre-miRNAs, by modulating the equilibrium of the RAN/RANBP1/RANGAP1 axis, the main regulator of nucleo-cytoplasmic transport. The levels of pre-miRNAs and mature miRNAs were assessed by qRT-PCR, in total extracts and after differential nuclear/cytoplasmic extraction. RANBP1 expression is the limiting step in the regulation of SGK1-SP1 dependent nuclear export. These results were validated in unrelated tumor models and primary human fibroblasts and corroborated in tumor-engrafted nude mice. The levels of pri-miRNAs, DROSHA, DICER and the compartmental distribution of XPO5 were documented. Experiments using RANGTP conformational antibodies confirmed that SGK1, through RANBP1, decreases the level of the GTP-bound state of RAN. This novel mechanism may play a role in the epigenomic regulation of cell physiology and fate.

The serum- and glucocorticoid-regulated kinase 1 (SGK1) is a serine/threonine kinase belonging to the AGK kinase family and shares structural and functional similarities with AKT, PKC and S6K^1^,^2^,^3^. SGK1 regulates ion channels and carriers^4^,^5^, pumps^6^, enzymes^7^,^8^, and transcription factors^9^,^10^,^11^,^12^ and mediates growth factor-dependent cell survival and growth signals^5^. SGK1 is regulated by mTOR-dependent phosphorylation of the hydrophobic motif (H-motif) on serine 422^13^, followed by phosphorylation and full activation by 3-phosphoinositide-dependent kinase-1 (PDK1)^14^. SGK1 is regulated by cAMP^15^, insulin^7^,^15^,^16^,^17^,^18^,^19^,^20^, glucocorticoids^4^, IL-2^21^, IGF-1^22^, and TGFβ^23^ and promotes survival and proliferative signals in normal and cancer cells. SGK1 expression and/or activity is increased in several human tumors, including breast^24^,^25^, tongue^26^, ovarian^27^ and prostate^28^ cancer, multiple myeloma^29^ and non-small cell lung cancer^30^. SGK1 knock-out models have shown resistance to chemical carcinogenesis^31^. SGK1 regulates cell survival, proliferation and differentiation via the phosphorylation of Mouse Double Minute 2 (MDM2), leading to p53 ubiquitylation and proteosomal degradation^32^. Moreover, SGK1 influences mitotic stability in colon carcinoma cells by regulating the expression of RANBP1, the principal regulator of the RAN GTPase. SGK1 enhances the transcription of RANBP1 via SP1 activation and phosphorylation of serine 59, thus affecting Taxol sensitivity in these cells^10^. Recently, we screened a family of dual SRC/ABL small molecule inhibitors characterized by a substituted pyrazolo[3,4-d]pyrimidine scaffold for their ability to inhibit SGK1 and AKT1 kinase activity, competing with ATP for its binding domain^33^. Among these molecules, SI113 was found to be particularly selective for the inhibition of SGK1 kinase activity but weakly effective toward AKT1. In fact, the dose-dependent curve of SI113-dependent SGK1 and AKT1 inhibition showed that inhibition of SGK1 activity occurs with an IC50 value of 600 nmol/L, with a selectivity almost 1000-fold higher than that for AKT1^33^. Similarly, SI113 shows poor inhibition of other kinases (e.g., ABL and SRC) that were originally considered to be putative targets of SI113^34^.

Additionally, SI113 induces cell death and alters the growth rate of various malignant cell lines. SI113 induces apoptosis in RKO colon carcinoma cells when used either alone or in combination with paclitaxel^34^ and inhibits tumor growth in hepatocarcinoma models *in vitro* and *in vivo*^35^ and in glioblastoma cell lines, showing a synergistic effect with radiotherapy^35^. Proteome-wide biochemical analyses have shown that SI113 down-regulates the levels of downstream targets of SGK1 with defined roles in neoplastic transformation, such as MDM2, NDRG1 and RAN network members^36^. In recent years, the Ran network (e.g., RAN-GTP/GDP, RANGAP1 and RANBP1) has been postulated to be involved in the control of mitotic stability^10^,^37^, chromosome segregation^38^ and proliferation^39^ and in resistance to various drugs that function in pro-metaphase^10^,^40^. However, during interphase, this protein complex is distributed along a steep gradient of the GTP/GDP ratio at the nuclear pore and regulates the nuclear import/export of proteins and ribonucleic acids^41^,^42^. This system involves the function of specialized proteins (importins and exportins), which are also essential for the nuclear transport and extranuclear maturation of microRNA precursors^43^. The RAN:GTP:Exportin*-*5 molecular complex is now considered to be the engine for pre-miRNA export^44^,^45^. In particular, the pre-miRNA/XPO5/RANGTP complex migrates to the cytoplasm, where pre-miRNAs are released in response to the hydrolysis of RANGTP to RANGDP, stimulated by RANGAP1 and other required cofactors^43^,^46^. It has recently been demonstrated that SGK1 inhibition by GSK 650394 impairs the nuclear export of influenza vRNPs into the cytoplasm of A549 cells^47^. Nevertheless, the mechanisms by which SGK1 regulates the nuclear export of ribonucleoproteins remain to be elucidated. In the present study, we demonstrate for the first time that SGK1, acting through SP1-dependent transcriptional and functional regulation of RANBP1 and RANGAP1, affects the binding status of GTP/GDP of RAN as well as RANBP1/RANGAP1 protein abundance and the localization of XPO5, thus enhancing the nuclear export and maturation of pre-miRNAs, without significantly affecting pri-miRNA levels, in a cellular model of HCC and in several neoplastic and non-tumoral primary cell lines. This process is reversed either by SI113-dependent or sh-mediated SGK1 inhibition/depletion. This SGK1-dependent regulation of pre-miRNAs was further demonstrated *in vivo* by SI113 treatment of human HCC tumors xenografted in NOD/SCID mice. We defined a hierarchical model by which SGK1 modulates the RANBP1/RANGAP1-dependent regulatory activity of RAN, a pivotal regulator of the nucleus-to-cytoplasm transportation of pre-microRNAs. Moreover, we provided additional data supporting a crucial role of RANBP1 in the mechanism of action of SI113, a novel SGK1 inhibitor showing anticancer properties in several neoplastic models.

## Results

### Effects of SGK1 modulation on pre/mature-miRNA conversion in a hepatocarcinoma cell model

**Figure 1:**
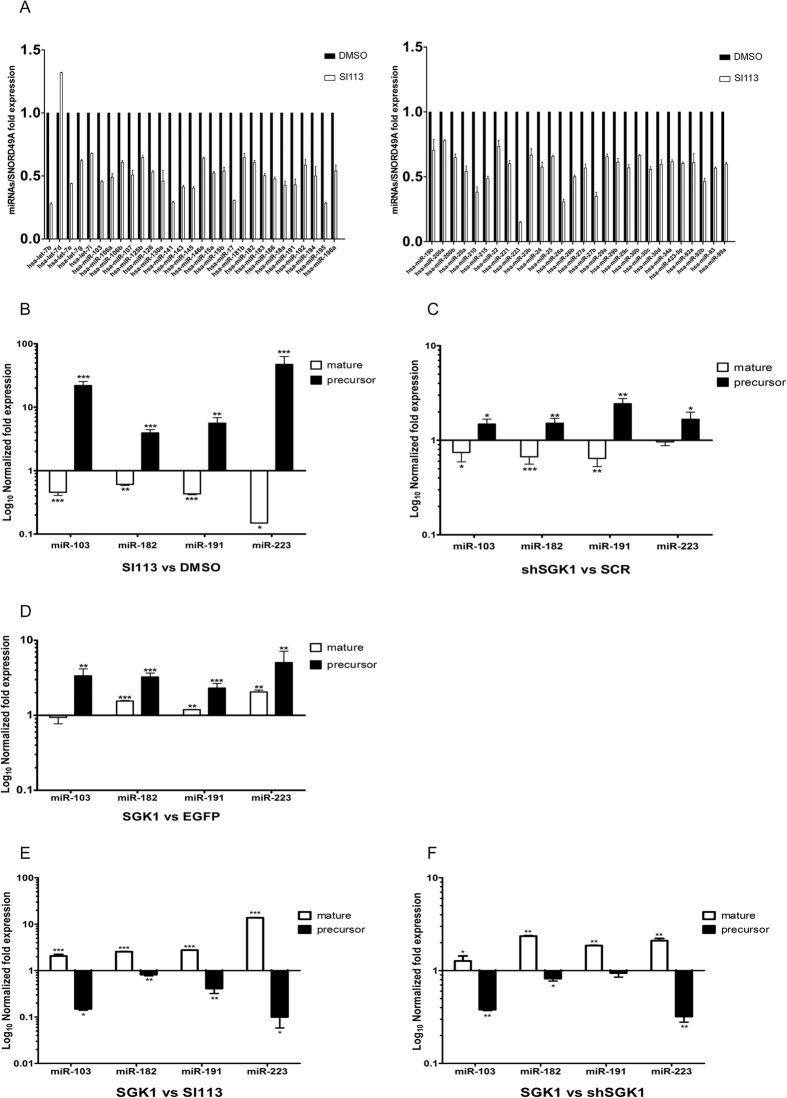
SGK1 modulation affects both mature and precursor microRNA levels. (**A**) Quantitative RT-PCR analysis of mature microRNA levels in HUH7 cells treated with either SI113 (12.5 μM for 72 h) or DMSO. One hundredth of the total amount of cDNA synthesized from 50 ng of total RNA was amplified via real-time PCR with 2X SYBR^TM^ Green master mix (Exiqon) in a miRCURY LNA^TM^ Cancer Focus microRNA PCR Panel 96-well plate, V1. MI (Exiqon). Values, calibrated by inter-plate calibrator and normalized to SNORD49A, are expressed as fold expression ± SD of quadruplicate samples and analyzed by *t*-test. (**B**) Quantitative RT-PCR analysis of four microRNAs (hsa-miR-103a, hsa-miR-182, hsa-miR-191 and hsa-miR-223), in their mature and precursor forms, in SI113-treated HUH7 cells and HUH7 control cells. Mature microRNAs were amplified using specific miRCURY LNA^TM^ PCR primer sets (Exiqon). For the precursors, 1 μg of total RNA was subjected to reverse transcription using the High Capacity cDNA Reverse Transcription Kit (Applied Biosystems) according to the instructions described in ref. 68, with a 1 μM antisense primer concentration (Table 1)^67^. Amplification was performed as reported in the Methods. The results, normalized to SNORD49A, are represented as Log_10_ of fold expression ± SD of triplicate assessments and evaluated using the *t*-test. (**C**) Quantitative RT-PCR analysis of mature and precursor microRNAs in stably silenced SGK1-HUH7 cells through lentiviral transduction. The assay was performed as above. (**D**) Quantitative RT-PCR analysis of mature and precursor microRNAs in HUH7 cells stably over-expressing EGFP-SGK1 through lentiviral transduction. The assay was performed as above. (**E-F**) Quantitative RT-PCR of mature and precursor microRNAs in HUH7 cells stably over-expressing EGFP-SGK1 and HUH7 cells treated with 12.5 μM SI113 for 72 h or stably silenced for SGK1, respectively. The results are expressed as Log_10_ of fold expression after subtraction of the relative control and are presented as the ratio of the values observed in SGK1-expressing cells to those in cells treated with 12.5 μM SI113 for 72 h or stably silenced for SGK1. Statistical significance is indicated at the top of the graph. *P ≤ 0.05; **P ≤ 0.01; ***P ≤ 0.001. Results are the average ± SD of three independent experiments each run in triplicates.

To verify whether SGK1 modulation affects the nuclear export of short non-coding RNAs (miRNAs) within the RAN:GTP:Exportin*-*5 complex^44^ by modulating the activity of the RAN/RANBP1 pathway^10^,^45^, we first used qRT-PCR to examine the levels of 85 microRNAs involved in cancer in a hepatic carcinoma-derived HUH7 cell line treated with either SI113 or vehicle alone (DMSO). SGK1 inhibition resulted in a significant decrease in the expression of 57 microRNAs, with the relevant exception of hsa-let-7d, which displayed enhanced expression (Fig. 1A), no significant variation was observed for the other microRNAs examined (data not shown). To assess whether the observed effect was attributable to a regulation of pre/mature-miRNA conversion rather than to a change in the transcriptional modulation of miRNAs, we selected 4 miRNAs (miR-103a, miR-182, miR-191, miR-223)^48^,^49^,^50^,^51^,^52^,^53^ playing different roles in the development of hepatocellular carcinoma and evaluated the relative expression levels of each pre-miRNA compared with the expression in the corresponding mature form (Fig. 1B). Among the examined miRNAs, a large increase in pre-miRNA expression was observed, whereas the corresponding mature forms appeared to be completely down-regulated upon SGK1 inhibition. Similar were obtained in HUH7 cells stably silenced for SGK1 (Fig. 1C), thus demonstrating the specificity of the SGK1 inhibition (Supplementary Figs 1b and 2c) in regulating the levels of precursor and mature forms of the tested miRNAs. The up-regulation of SGK1 expression (Supplementary Fig. 1a) via a lentiviral-mediated approach (Fig. 1D) resulted in a different expression pattern (72 h after infection), characterized by a net increase in both the mature and precursor miRNA forms. In an independent set of experiments, we compared the expression patterns in SI113-treated cells or stably silenced SGK1-HUH7 cells (shSGK1) with those in SGK1-over-expressing cells (Fig. 1E and F). Each condition was normalized to the orthologue control (i.e., vehicle alone, Scrambled, EGFP, respectively). In SGK1-over-expressing HUH7 cells, the expression of miRNA precursors was clearly down-regulated, whereas the expression of mature miRNAs appeared to be up-regulated compared with that in SI113-treated cells or shSGK1-HUH7 cells, suggesting that SGK1 activity is essential and limiting for the conversion of the pre/mature forms of the examined miRNAs.

### SGK1 inhibition affects pre-miRNA/miRNA conversion in several neoplastic and normal primary cell lines

**Figure 2:**
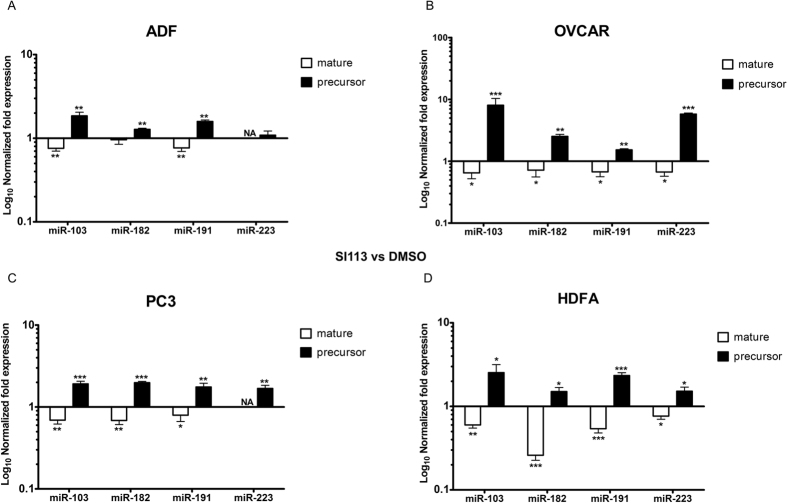
SI113-dependent SGK1 inhibition modulates the pre-miRNA/miRNA ratio in both neoplastic and primary normal cell lines. Quantitative RT-PCR analysis of four microRNAs (hsa-miR-103a, hsa-miR-182, hsa-miR-191 and hsa-miR-223), comprising both their mature and precursor forms, in the ADF (**A**), OVCAR3 (**B**), PC3 (**C**) and primary HDFa (**D**) cell lines, treated with either SI113 (12.5 μM for 72 h) or DMSO. qRT-PCR was performed as previously described, and the results for both forms were normalized to SNORD49A and are presented as Log_10_ of fold expression ± SD of triplicates. Statistical significance was determined using the *t*-test and is reported at the top of the graph. *P ≤ 0.05; **P ≤ 0.01; ***P ≤ 0.001. Results are the average ± SD of three independent experiments each run in triplicates.

We extended our approach to several cancer cell lines: ADF (human glioblastoma cell line), PC3 (human prostate cancer cell line) and OVCAR3 (human ovarian carcinoma cell line). In all of the examined cell lines, the results of SI113-dependent SGK1 inhibition recapitulated the results obtained in HUH7 cells, i.e., an increase in precursors and a net reduction in the mature forms of the examined miRNAs (Fig. 2A–C). Interestingly, the same results were obtained in primary non-tumoral cell lines (HDFA-human dermal fibroblast) (Fig. 2D), suggesting a general role for SGK1 in the cellular physiology of miRNA nucleo-cytoplasmic transport.

### SGK1 is essential and rate limiting in the control of precursor miRNA nuclear export

**Figure 3:**
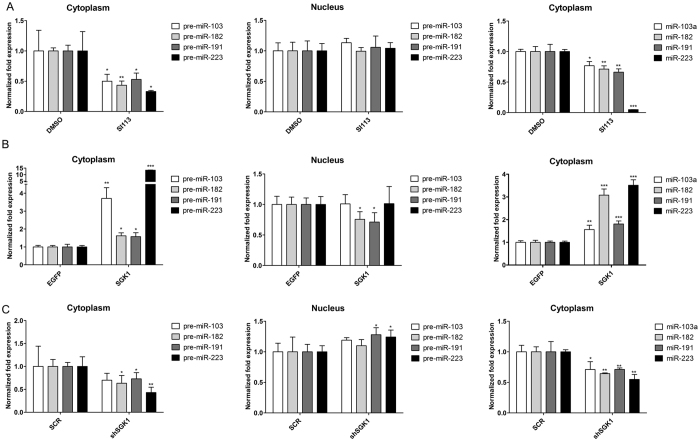
SGK1 over-expression or inhibition regulates nuclear and cytoplasmic precursor and mature microRNA levels. (**A**) Quantitative RT-PCR analysis of precursor and mature microRNAs in the nuclear and cytoplasmic fractions of SI113-treated HUH7 cells. Isolation and purification of both cytoplasmic and nuclear RNA from cells was conducted using a specific kit (NorgenBiotek Corp., Thorold, ON, Canada). To avoid genomic DNA contamination, especially in the nuclear fraction, DNase I (TURBO^TM^ DNase, Ambion, Life Technologies, Paisley, UK) digestion was performed following the manufacturer’s instructions. RT-PCR was performed as previously described. (**B**) Quantitative RT-PCR analysis of precursor and mature microRNAs in the nuclei and cytoplasm of HUH7 cells stably over-expressing SGK1. The assay was performed as described above. (**C**) Quantitative RT-PCR analysis of precursor and mature microRNAs in the nuclei and cytoplasm of HUH7 cells stably silenced for SGK1. The assay was performed as described above. Statistical significance is reported at the top of the graph. *P ≤ 0.05; **P ≤ 0.01; ***P ≤ 0.001. Results are the average ± SD of three independent experiments each run in triplicates.

To verify whether the effects of SGK1 on pre/mature miRNA conversion were dependent on active regulation of precursor nuclear export, we performed differential nuclear and cytoplasmic RNA extraction. The levels of precursor and mature miRNAs were assessed in both compartments via qRT-PCR. We found that SI113-dependent SGK1 inhibition resulted in a dramatic reduction of the cytoplasmic expression of both pre-miRNAs and mature miRNAs, whereas no significant changes in the levels of nuclear precursors were recorded (Fig. 3A). Conversely, SGK1 over-expression (Fig. 3B) (Supplementary Fig. 1a) caused cytoplasmic accumulation of precursors (72 h after infection), with a relative increase in their mature forms and a slight but significant decrease in the expression of nuclear precursors at least in two of the four miRNAs considered. In order to verify the specificity of our model, a similar differential nucleus/cytoplasm separation was conducted in HUH7 cells stably silenced for SGK1. The effect of SGK1 silencing was similar to that observed with SI113 treatment: a sharp and dramatic reduction in the cytoplasmic levels of both pre-miRNAs mature miRNAs and a nuclear accumulation of pre-miRNAs at list in two of the four miRNAs considered (Fig. 3C). Bona fide nuclear extraction was confirmed by the absence of detectable mature miRNAs in the nucleus, whereas the small nucleolar RNA SNORD49A was easily detected and amplified. Taken together, these data strongly suggest that SGK1 expression and activity influence the export and, indirectly, the maturation of the examined miRNAs in HUH7 cells, probably by regulating the RAN system.

### SGK1 regulates pre-miRNA nuclear export in a RANBP1-dependent manner by signaling through SP1

**Figure 4:**
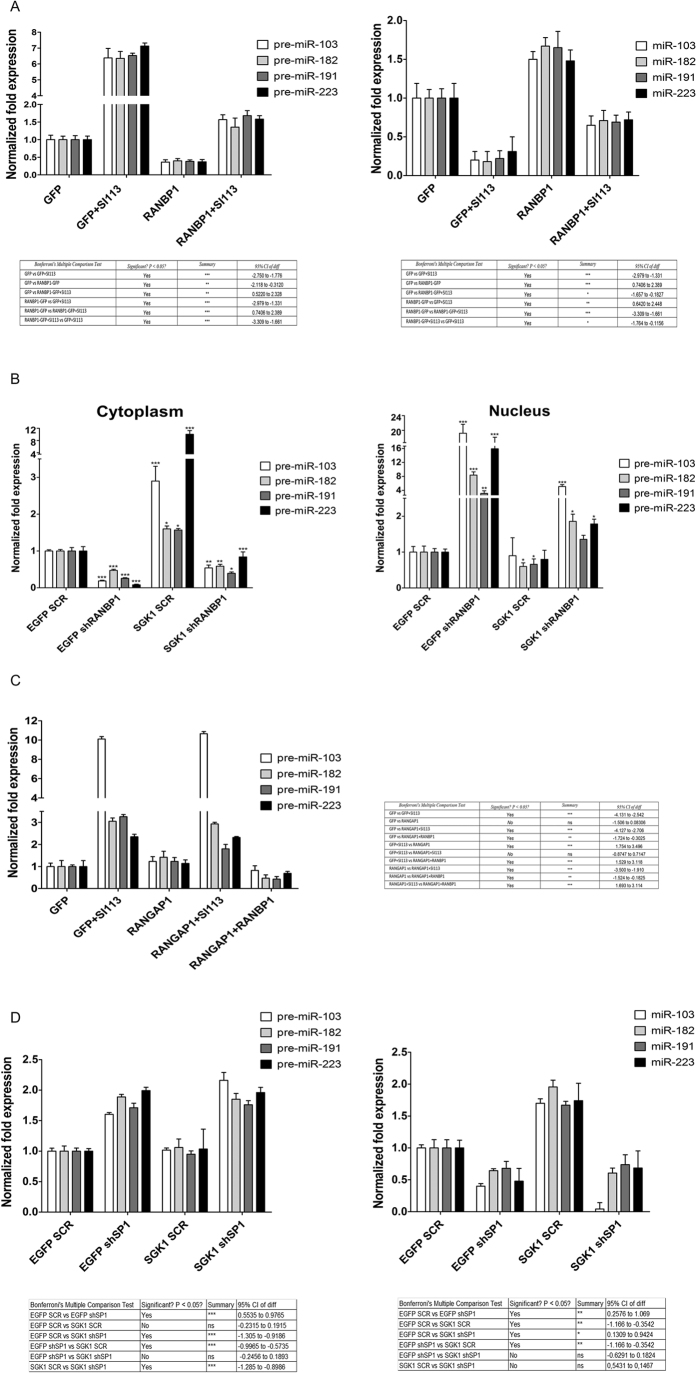
SGK1 regulates pre-miRNA nuclear transport via the SP1/RANBP1/RANGAP1 axis. (**A**) Quantitative RT-PCR analysis of precursor and mature microRNAs in HUH7 cells over-expressing GFP-RANBP1 or GFP, with or without 12.5 μM SI113 treatment for 72 h, compared with the GFP control cell line. (**B**) Quantitative RT-PCR analysis of precursor microRNAs in the nuclei and cytoplasm of HUH7 cells stably over-expressing EGFP-SGK1 or EGFP, with or without transiently silenced RANBP1 (shRANBP1). (**C**) Quantitative RT-PCR analysis of four precursor microRNAs (hsa-miR-103a, hsa-miR-182, hsa-miR-191 and hsa-miR-223) in HUH7-RANGAP1 cells, with or without SI113 treatment (12.5 μM for 72 h), and HUH7-RANGAP1/RANBP1 cells compared with control cells, with or without of the same treatment. (**D**) Quantitative RT-PCR analysis of precursor and mature microRNAs in HUH7 cells stably over-expressing EGFP-SGK1 or EGFP, with or without transiently silenced SP1 (shSP1), compared with the EGFP/SCR control cell line. The results are plotted as the fold expression and were analyzed via one-way analysis of variance (ANOVA), followed by the Bonferroni mean test for multiple comparisons. The assays were performed as described above. *P ≤* *0.05; **P ≤* *0.01; ***P ≤* *0.001. Results are the average ± SD of three independent experiments each run in triplicates.

The observed effects could be attributed to the SGK1-dependent regulation of RANBP1 and RAN^10^,^54^ expression, affecting the nuclear export of miRNAs^41^. To substantiate our hypothesis, we obtained RANBP1 over-expressing cells through transient transfection (Supplementary Fig. 1d) and assessed the levels of precursor and mature forms of miRNAs either in the presence or absence of SI113 (12.5 μM, 72 h). We found that RANBP1 over-expression down-regulated the levels of miRNA precursors and antagonized the effect of SI113 to some degree when used in combination (Fig. 4A, left panel). Interestingly the expression pattern of mature miRNAs under the same conditions was perfectly reversed in the presence of SI113, causing significant down-regulation of these forms, whereas RANBP1 enhanced the levels of mature miRNAs when used as a single agent and antagonized the effects of SI113 to some degree when used in combination (Fig. 4A, right panel). The results of RANBP1 silencing (Supplementary Fig. 1e and 2b) mirrored the effects of RANBP1 over-expression, leading to a significant reduction in the expression of mature miRNAs, which was antagonized by SGK1 over-expression to some degree. Indeed, differential nuclear and cytoplasmic extraction showed that RANBP1 silencing resulted in a consistent reduction of the levels of miRNA precursors in the cytoplasmic compartment and high nuclear accumulation of these molecules; these effects were partially antagonized by SGK1 over-expression (Fig. 4B), which probably enhanced residual RANBP1 expression. The cytoplasmic expression of mature miRNAs was modified by RANBP1 silencing as expected. The levels of mature miRNAs decreased significantly upon RANBP1 silencing, an effect only in part antagonized by the concomitant SGK1 overexpression (Supplementary Fig. 3). Taken together, these data strongly suggest that RANBP1 mediates the SGK1 dependent control of nuclear export and conversion of pre-miRNAs. Notably, this effect was specific to RANBP1, as over-expression of RANGAP1 (Supplementary File 1d), the RANBP1 interactor in the RAN-GTPase complex^55^, had no measurable effect on precursor/mature miRNA conversion in the absence of RANBP1 over-expression (Fig. 4C). Similarly, RANBP1 was able to rescue HUH7 cells from the anti-proliferative effect exerted by SI113 (12.5 μM, 72 h), confirming our previous observations^10^ (Supplementary Fig. 4a). In contrast, RANGAP1 over-expression did not counteract the effect of SI113 unless RANBP1 was also over-expressed (Supplementary Fig. 4b). In our previous work^10^, we showed that SGK1 was able to regulate RANBP1 and, indirectly, the function of RAN through serine 59 phosphorylation of SP1, which is the main RANBP1 transcriptional regulator. To determine whether SGK1 modulates the nuclear transport of pre-miRNAs through SP1 and RANBP1, we performed specific silencing of SP1 in the presence or absence of SGK1 over-expression (Fig. 4D). SP1 silencing (Supplementary Fig. 2a) caused an increase in the miRNA levels of precursors and a reduction of the mature forms, similarly to what obtained by SI113-dependent SGK1 inhibition. In addition, under SP1 silencing, over-expression of SGK1 was unable to counteract the effects of SP1 silencing on the nuclear export of pre-miRNAs, suggesting that SP1 is necessary for this particular SGK1 dependent activity (see above).

### SGK1 inhibition affects the expression of RANBP1/RANGAP1, causing changes in miRNA nuclear transport in HCC tumors engrafted in NOD/SCID mice

**Figure 5:**
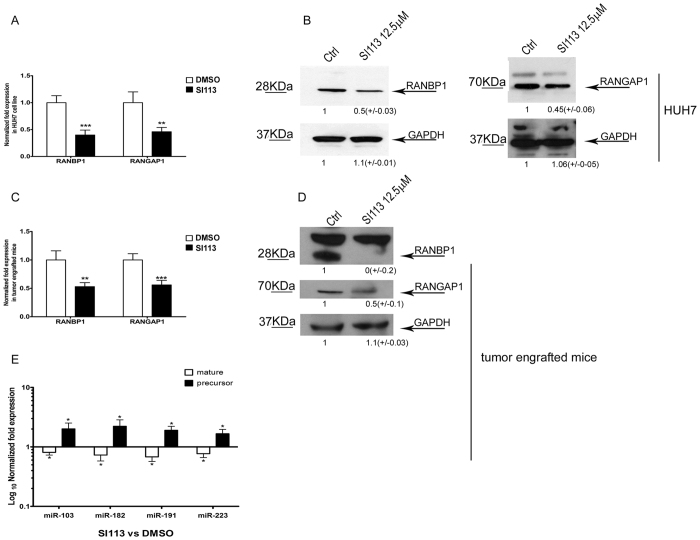
SI113-dependent SGK1 inhibition affects RANBP1 and RANGAP1 transcription, influencing mature and precursor microRNA levels both *in vitro* and *in vivo*. (**A**) Quantitative RT-PCR analysis of RANBP1 and RANGAP1 mRNA levels in SI113-treated HUH7 cells compared with control cells. The results, normalized to the HPRT housekeeping gene, are represented as the fold expression ± SD of triplicate assessments and were evaluated using the *t*-test. (**B**) Western blot analysis of proteins from HUH7 cells with or without 12.5 μM SI113 treatment for 72 h. Cell extracts (50 μg aliquots) were assessed via SDS-polyacrylamide gel electrophoresis, followed by immunoblotting using a RANBP1 antibody, a RANGAP1 antibody and a GAPDH antibody. (**C**) Quantitative RT-PCR analysis of RANBP1 and RANGAP1 mRNA levels in tumor tissues of HCC xenografts in NOD/SCID mice with or without SI113 treatment. The HCC mouse xenograft model and treatment regimen were developed as previously reported^54^. (**D**) Western blotting analysis of proteins from mouse tumor tissues with or without SI113 treatment. (**E**) Quantitative RT-PCR analysis of both mature and precursor microRNAs in mouse tumor tissues with or without SI113 treatment. The values are plotted as Log_10_ of fold expression ± SD and evaluated using the *t*-test. The assay was performed as described above. Statistical significance is reported at the top of the graph. *P ≤ 0.05; **P ≤ 0.01; ***P ≤ 0.001. Cropped gels are shown and full-length gels are included in SFs. Results are the average ± SD of three independent experiments each run in triplicates.

SI113-dependent inhibition of SGK1 in HUH7 cells results in the reduction of RANBP1 expression, as detected through assessments of RNA and protein via qRT-PCR and/or WB, respectively (Fig. 5A,B). Similar results were obtained in HUH7 tumors engrafted in NOD/SCID mice treated with either SI113 or vehicle alone^54^ (Fig. 5C,D). In this set of experiments, we noted, beside RANBP1, RANGAP1 expression was also controlled by SGK1. In fact, SI113-dependent SGK1 inhibition down-regulated RANBP1 and RANGAP1 RNA and protein levels in HUH7 cells grown in culture (Fig. 5A,B) or engrafted in NOD-SCID mice (Fig. 5C,D). Moreover, the expression of all the tested miRNA precursors was significantly increased, whereas the mature form were reduced by SI113 in HUH7 cells engrafted in NOD-SCID mice (Fig. 5E), confirming the findings obtained in HUH7 cells in culture. We conclude that SGK1 is a pivotal regulator of both RANBP1 and RANGAP1 expression and modulates nuclear export thus affecting miRNA conversion/transport.

### SGK1 is a pivotal regulator of the GTP/GDP status of RAN through RANBP1/RANGAP1 modulation and affects the GTP nuclear gradient and export efficacy

**Figure 6:**
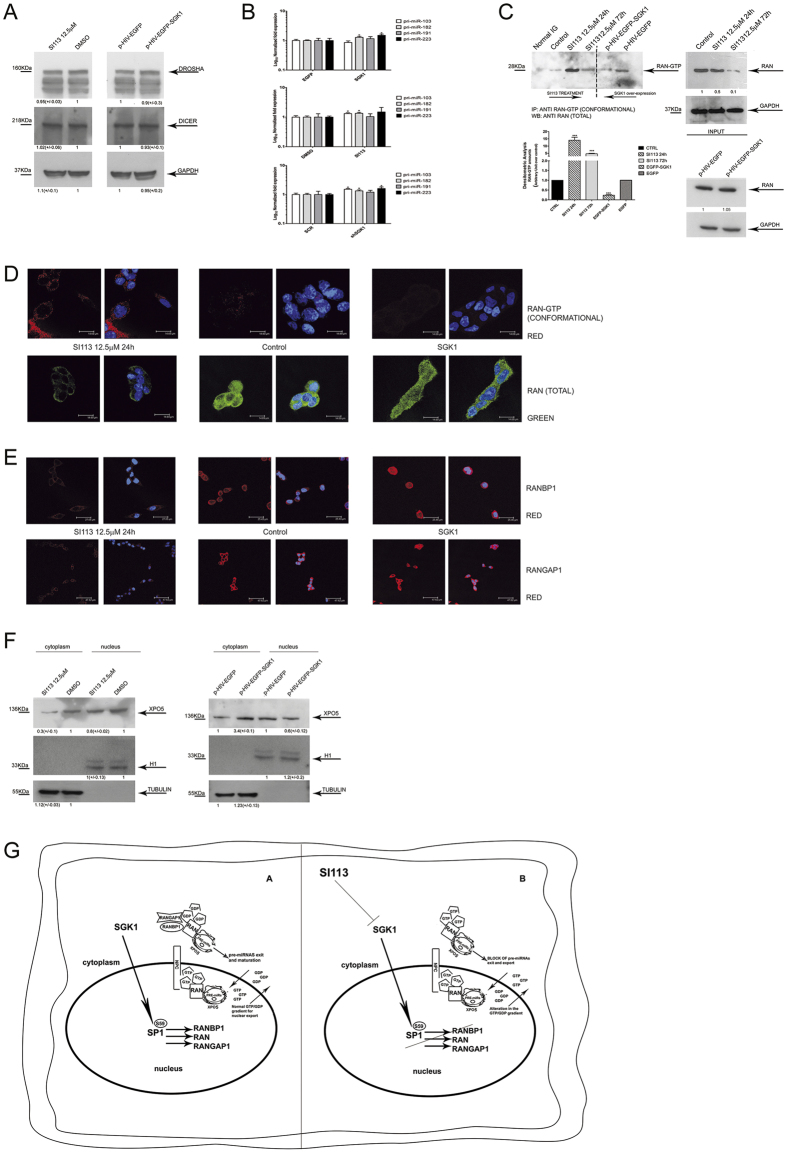
SGK1 fluctuations disrupt the equilibrium of the RAN/RANBP1/RANGAP1 axis, affecting the GTP gradient and nuclear transport. (**A**) Western blot of proteins from HUH7 cells, treated with SI113 (12.5 μM; 72 h) or vehicle (left), or transduced with EGFP-SGK1 or EGFP (right). Cell extracts were separated, blotted and decorated by DROSHA, DICER and GAPDH antibodies. (**B**) Quantitative RT-PCR analysis of pri-microRNAs in HUH7 cells stably over-expressing EGFP-SGK1 or EGFP (upper panel), in SI113-treated HUH7 and control cells (middle panel), in shSGK1 and shScrl HUH7 cells (bottom panel). The assay was performed as above. Results are represented as Log_10_ of fold expression ± SD and evaluated using *t*-test. (**C**) Immunoprecipitation of RAN-GTP from HUH7 cells treated with SI113 (12.5 μM; 24 h and 72 h) or vehicle or transduced with a lentivirus encoding p-HIV-EGFP or p-HIV-EGFP-SGK1. Immunoprecipitation was performed as described in methods. The immunoprecipitates (left) and the corresponding total extracts (right) were blotted and decorated by anti-Ran total goat polyclonal antibodies. GAPDH was used as a loading control (right). (**D**) Representative immunofluorescence analysis of HUH7 cells either treated with SI113 (12.5 μM) or over-expressing SGK1 (pcDNA4-TO-SGK1) compared with control cells. Samples were incubated with either a conformational Active-RAN monoclonal antibody or with an anti-Ran total goat polyclonal antibody and DAPI (0.05% μg/ml^1^). Since no variation in fluorescence was observed between the different experimental controls; only one control is presented in the panel. (Leica TC SP2 microscope with a x63 objective and Leica confocal software). (**E**) HUH7 cells treated as above, were stained with either an anti-RANBP1 goat polyclonal antibody or an anti-RANGAP1 monoclonal antibody and DAPI (0.05% μg/ml). (**F**) HUH7 cells treated with SI113 (12.5 μM; 72 h) or vehicle (left) or transduced with EGFP-SGK1 or EGFP. After nuclear/cytoplasmic separation, solubilized extracts (50 μg and 25 μg for the cytoplasmic and nuclear fractions, respectively) were separated by SDS-polyacrylamide gel electrophoresis, decorated by XPO5, α-Tubulin, H1 antibodies. (**G**) Schematic outline of the SGK1-dependent pre-miRNA nuclear transport mechanism. Cropped gels are shown and full-length gels are included in SFs. Results are the average ± SD of three independent experiments each run in triplicates.

Pre-miRNAs are subjected to dynamic conversion and processing mediated by DROSHA and DICER that might have a role in the observed alterations in the amounts and maturation of pre-miRNAs. We checked the expression of these proteins through western blot analysis in presence of either SI113-dependent SGK1 inhibition or SGK1 over-expression. There were no variations in DROSHA or DICER expression under these conditions (Fig. 6A). To rule out a possible transcriptional effect on miRNA expression, the expression of pri-miRNAs was evaluated in SGK1 over-expressing as well as in SGK1 silenced cells or treated with SI113. In all these conditions we were unable to detect significant variations in the expression of pri-miRNAs. The small changes observed, if any, were not related to a specific genetic or pharmacologic manipulation (Fig. 6B). We conclude that transcriptional effects cannot be taken in account to explain the SGK1 dependent effects on miRNAs transport and maturation. The SGK1-dependent regulation of RANBP1 and RANGAP1 protein expression is expected to produce a measurable impact on the GTP/GDP-bound status of RAN. We used a RAN-GTP-specific conformational antibody and a Ran-specific antibody to immunoprecipitate RANGTP and RAN, respectively, in the presence of either SI113-dependent SGK1 inhibition or SGK1 over-expression. SGK1 inhibition resulted in an increase in the amount of GTP-bound RAN in a time-dependent manner, with a peak at 24 h after the addition of the inhibitor. Conversely, exogenous SGK1 over-expression reduced the amount of GTP-bound RAN (Fig. 6C). These results were confirmed by immunofluorescence experiments using HUH7 cells. SI113 treatment increased the amount of accumulated GTP-bound RAN in the perinuclear cytoplasm, whereas the abundance of total RAN was dramatically reduced, suggesting that most of the RAN was in the GTP-bound form when SGK1 was inhibited. In control cells, the GTP-bound form was present at much lower levels, and its distribution was mostly nuclear. A characteristic that was even more evident in cells over-expressing SGK1 where most of the RAN was in the GDP-bound form (Fig. 6D and Supplementary Fig. 5a and b). As expected, treatment with SI113 completely abolished the cellular staining of RANBP1 and RANGAP1, causing complete cytoplasmic depletion, whereas over-expression of SGK1 induced a significant up-regulation of both proteins, with strong cytoplasmic accumulation (Fig. 6E and Supplementary Fig. 5a and b).

We also expected to see changes in the localization and amount of Exportin-5, the final carrier in pre-miRNA nuclear transport, in association with the RAN network. Indeed, in HUH7 cells treated with SI113, XPO5 was predominantly detected in the nuclear compartment, whereas in SGK1 over-expressing HUH7 cells, XPO5 was mainly detected in the cytoplasm (Fig. 6F).

## Discussion

Facilitated transport through karyopherins requires energy provided by RAN-GTP-binding proteins, which function as molecular motors that alternate from an active GTP-bound state to a GDP-bound state^56^. RANBP1 is the principal regulator of the RAN GTPase: it cooperates with RANGAP1 (RAN GTPase-activating protein) to promote GTP hydrolysis by RAN; in addition, RANBP1 has been found to bind to RANGTP, thus regulating its association or dissociation from effectors of the family of nuclear transport receptors (for example, Exportin 5/XPO5, Importin Beta and Exportin1/CRM1)^44^,^45^,^55^,^57^. RANBP1 is a key regulator of RAN-dependent signals in all downstream-regulated events, including interphase nuclear import and export, mitotic spindle formation and nuclear post-mitosis^58^,^59^,^60^,^61^. Like other members of the RAN network, RANBP1 is transcriptionally regulated during the cell cycle. The transcriptional control of RANBP1 is dependent on E2F- and SP1-regulated promoter elements^62^. The perturbation of cell cycle-regulated RANBP1 transcription affects mitotic processes: RANBP1 over-expression leads to multipolar spindles and fragmented centrosomes^58^, whereas RANBP1 down-regulation facilitates microtubule stabilization, impairing dynamic spindle activity^37^,^38^. RANGAP1 and RANBP1 are mainly localized in the cytoplasm, where they function to create a RANGTP gradient that is necessary to control the effectiveness and the directionality of nuclear transport, with a higher concentration of RANGTP in the nucleus and a higher concentration of RANGDP in the cytoplasmic compartment^56^,^63^,^64^. In the present report, we have demonstrated that SI113-dependent SGK1 inhibition induces a generalized down-regulation of miRNA expression in hepatocellular carcinoma cells. This phenomenon results from the reduced conversion of miRNA precursors into mature miRNAs, which is dependent on the amount of RANBP1 protein that is available in cells. Since this phenomenon has been observed in cancer cell lines as well as in a noncancerous primary fibroblast cell line, SGK1 and RANBP1 may participate in the general physiologic control of miRNA transport and maturation, increasing the overall impact and biological value of the data. The general control of conversion and pre-miRNA nuclear transport appears to be dependent on the cellular level of RANBP1. All of the evidence gathered herein indicates that the effects of SGK1 on pre-miRNA nuclear export and maturation require the presence of SP1 and RANBP1. This mechanism is mediated by SGK1, as evidenced by the results of chemical inhibition, silencing and over-expression.

SI113-dependent inhibition of SGK1, as well as the SGK1 silencing, leads to an impressive decrease in the cytoplasmic levels of both precursor and mature miRNAs, a condition that is perfectly mirrored by SGK1 over-expression. RANBP1 over-expression greatly facilitates the conversion of precursor to mature miRNAs, an effect that is antagonized by SI113 treatment to some degree, suggesting that the abundance of RANBP1 is the limiting factor in this process. In contrast, RANGAP1 does not appear to play a limiting role, as when RANGAP1 is over-expressed, it does not affect either the expression of miRNA precursors or the activity of SI113. Our data suggest that RANBP1, a trans-activator of RANGAP1 activity, is upstream and limiting for the function of RANGAP1, although RANGAP1 appears to be modulated and mis-localized in response to SGK1 fluctuations in parallel with RANBP1. The entire system, as expected^10^, is under SP1 transcriptional control. SP1 silencing results in a complete reversal of SGK1-dependent pre-miRNA nuclear transport regulation. Interestingly, the amount of RANBP1 appears to be a limiting also for the anti-proliferative effects of SI113. In support of these findings, differential RNA nuclear/cytoplasmic extraction suggested that SGK1 activity affects the transport of pre-miRNAs and, thus, their precise distribution between the nucleus and cytoplasm, with indirect effects on miRNA maturation. To corroborate this hypothesis, we present evidence that the modulation of SGK1 activity has no specific effect on the protein expression of DROSHA and DICER, neither on the pri-miRNAs amount, further suggesting a role for nucleo-cytoplasmic transport, rather than processing or transcription, in the changes in the pre-miRNA/miRNA ratio. In the present report, we also demonstrate that SGK1 activates the transcription of RANGAP1. As previously demonstrated for RANBP1^10^, this effect is likely also a consequence of SP1 phosphorylation. In fact, SGK1 activates and phosphorylates SP1 on serine 59, a regulator of nucleocytoplasmic trafficking-related genes^65^. The relevance of these findings was confirmed by *in vivo* results obtained using HCC xenografted in NOD SCID mice. SI113 treatment of these mice led to a significant decrease in the expression of RANBP1 and RANGAP1 and an alteration of the pre/mature miRNA levels, a bona fide marker of altered nuclear export. A more functional or mechanistic interpretation of our observations was tentatively provided by the results obtained through immunoprecipitation and immunofluorescence experiments using RANGTP conformational antibodies. SGK1 inhibition resulted in cytoplasmic accumulation of GTP-bound RAN, which was an expected consequence of the reduced expression of RANBP1 and RANGAP1. The cytoplasmic accumulation of GTP-bound RAN disrupts the RANGTP nucleo-cytoplasmic gradient, which is necessary for nucleo-cytoplasmic trafficking^56^, and interferes with the localization and release of pre-miRNAs bound to XPO5/RAN^45^,^66^ (Fig. 6G).

In conclusion, SGK1 inhibition is expected to significantly reduce nuclear transport through SP1-dependent reduction of the expression of RANBP1, RANGAP1 and RANGTP. However, tumors over-expressing SGK1 may be characterized by deregulated and inappropriate nuclear export that may eventually favor cell proliferation. Moreover, we postulate that the general regulation of pre-miRNA nuclear transport through SGK1 could provide new insights into our understanding of the epigenomic adjustments that affect cell metabolism and fate as a consequence of miRNA formation and turnover.

## Methods

### Cell lines

The human HCC HUH7, human embryonic kidney HEK293T and human prostate cancer PC3 cell lines were cultured in DMEM (Life Technologies, Inc., Grand Island, NY), the primary human dermal fibroblast HDFa, human ovarian carcinoma OVCAR3, and human glioblastoma ADF cell lines were cultured in RPMI-1640 (Life Technologies). Culture media were supplemented with 10% fetal bovine serum and a 1% penicillin-streptomycin solution (Aurogene, Rome, Italy). The cells were cultured at 37 °C in a humidified atmosphere of 5% CO_2_ and 95% air. Cell lines were obtained from the ATCC (Georgetown University in Washington, DC), excluding the ADF cell line, which was kindly provided by Dr. Marco Paggi (Regina Elena Cancer Institute, Rome, Italy).

### SI113 treatment

SI113 was used as previously reported^33^. The drug was diluted in dimethylsulfoxide (DMSO) at a 10 mM initial concentration and stored at −20 °C. Cells transfected with pRANBP1-GFP, GFP and pRANGAP1 or the empty vector were treated 6 h after transfection, cell pellets were collected after 72 h.

### Plasmids

Plasmids encoding pRANBP1-GFP, pGFP and pHHS10B-RANGAP1were kindly provided by Dr. Lavia P.^58^ and transfected into cells as reported elsewhere^10^. The sequences encoding wild-type *SGK1* were cloned into the pcDNA4-TO expression vector (Invitrogen, Milan, Italy) as previously described^21^. The coding sequence of wild-type *SGK1* within PcDNA4-TO was sub-cloned into the pHIV-EGFP expression vector (Addgene plasmid 21373) to obtain a self-inactivating lentiviral plasmid for co-expression of *SGK1* and EGFP, as described elsewhere^54^. p-HIV-EGFP-*SGK1* or pHIV-*EGFP* (negative control), as well as pLKO.1-puro-shSGK1 and shScrl (SIGMA SHC002V) were used to generate lentiviral particles in HEK293T packaging cells, as previously reported^54^. Silencing of RANBP1 and SP1 was achieved via shRNABP1 (sc-41848) or shSP1 (sc-29487-SH) plasmid transfection, respectively.

### Quantitative real-time PCR

RNA extraction was performed using the miRNeasy^®^ Mini Kit (Qiagen, Valencia, CA, USA) following the manufacturer’s instructions. Cytoplasmic and nuclear RNA were purified with a specific kit (#21000, NorgenBiotek Corp., Yhorold, ON, Canada) according to the manufacturer’s instructions and treated with DNase I (TURBO^TM^ DNase, Ambion, Life Technologies, Paisley, UK) as indicated in the protocol to avoid contamination by genomic DNA in the nuclear fraction. RNA was quantified using a Multiskan Go spectrophotometer (Thermo Scientific, Madison, WI, USA). The quality of RNA was assayed by determining the 260/280 absorbance ratio and through formaldehyde agarose gel electrophoresis. For mature microRNAs, 50 ng of total RNA was subjected to reverse-transcription with the miRCURY LNA^TM^ Universal cDNA synthesis kit (Exiqon, Vedbaek, Denmark), following the manufacturer’s instructions. One hundredth of the total amount of cDNA was amplified via real-time PCR using 2X SYBR^TM^ Green master mix (Exiqon) in a miRCURY LNA^TM^ Cancer Focus microRNA PCR Panel 96-well plate, V1.MI (Exiqon), following the manufacturer’s instructions. Each sample was evaluated in quadruplicate. Mature miR-103a, miR-182, miR-191, miR-223 and SNORD49A were amplified using a specific miRCURY LNA^TM^ PCR primer set (Exiqon). For the miR-103a, miR-182, miR-191, miR-223^67^ and SNORD49A precursors (pri-miRNAs and pre-miRNAs), 1 μg of total RNA was subjected to reverse transcription using the High Capacity cDNA Reverse Transcription Kit (Applied Biosystems, Foster City, CA, USA) according to the instructions reported previously^68^, but modifying the concentration of the antisense primer cocktail (listed in Table 1) to 1 μM. For the RANBP1, RANGAP1 and SGK1 mRNAs, 1 μg of total RNA was subjected to reverse transcription using the High Capacity RNA-to-cDNA Kit (Applied Biosystems, Foster City, CA, USA) according to the manufacturer’s instructions. One microliter of cDNA was amplified via real-time PCR using Promega SYBR green (Promega, Madison, WI, USA) and 10 pmol of specific primers (Tables 1 and 2).The obtained values were normalized to the house-keeping gene HPRT using specific primers^69^.

Real-time PCR assays were performed in triplicate in a total volume of 20 μl using a Bio-Rad iQ^TM^ 5 apparatus under the following conditions: initial denaturation step at 95 °C for 3 minutes, followed by 40 cycles of 10 s at 95 °C and 1 minute at 60 °C (for miRNA precursors) or 57 °C (for RANBP1 and RANGAP1 mRNAs). The specificity of the PCR products was determined through melting curve analysis.

### Nuclear and cytoplasmic protein extraction

Nuclear and cytoplasmic EGFP/SGK and SI113 proteins from treated or untreated HUH7 cell lines were extracted using the NE-PER kit (#78833, Thermo Scientific). The cells (1 × 10^6^) were washed with PBS (1X), and the pellet was re-suspended in CER-I buffer. After 10 minutes on ice, CER-II buffer was added to the sample. The re-suspended cell pellet was then centrifuged, and the supernatant was collected (cytoplasmic extract) and placed on ice. The nuclear pellet fraction was subsequently re-suspended in ice-cold NER buffer and placed on ice for 40 minutes. After washing (4 times) and centrifugation, the supernatant (nuclear extract) was recovered. Nuclear and cytoplasmic proteins were separated by western blot.

### Immunoblotting and immunoprecipitation

Cells were processed as indicated in a previous report^10^ and probed with a goat polyclonal RANBP1 antibody (sc-1160, Santa Cruz Biotechnology, Santa Cruz, CA, USA), a mouse monoclonal RANGAP1 antibody (sc-28322, Santa Cruz Biotechnology), a rabbit polyclonal GAPDH antibody (sc-25778, Santa Cruz Biotechnology), a mouse monoclonal DROSHA antibody (sc-393591, Santa Cruz Biotechnology), a rabbit polyclonal DICER antibody (sc-30226, Santa Cruz Biotechnology), a goat polyclonal antibody for total RAN (sc-1156 Sant Cruz), a goat polyclonal XPO5 antibody (sc-34619, Santa Cruz Biotechnology), a mouse monoclonal H1 antibody (sc-8030, Santa Cruz Biotechnology), a rabbit polyclonal α-TUBULIN antibody (sc-5546, Santa Cruz Biotechnology) and a rabbit polyclonal SP1 antibody (sc-14027, Santa Cruz Biotechnology) and a rabbit polyclonal SGK1 antibody (cat# 07-315 EDM Millipore Corporation, CA, USA).

Immunoprecipitation of the RAN-GTP form from HUH7 cell protein lysates was conducted using a pre-linked gamma-bound G-Sepharose (GE Healthcare 17-0885-01) conformational antibody recognizing the GTP-bound form of RAN (26915 NewEast Bioscience). After overnight incubation at 4 °C on a rotating wheel, samples were pre-cleared with normal mouse IG for 2 h at room temperature and washed. The immunoprecipitates were separated by gradient SDS-PAGE, transferred to nitrocellulose and decorated with a goat polyclonal antibody for total RAN (SC-1156 Santa Cruz). The corresponding input was loaded in the same gel and incubated with the same antibody or with a rabbit polyclonal GAPDH antibody as a loading control.

### Immunofluorescence

The immunofluorescence analysis of the RAN-GTP conformation was performed as follows. SI113-treated and control HUH7 cells as well as SGK1 and EGFP HUH7 cells grown on coverslips were washed with 1X PBS and permeabilized in 1% Triton-X100 PHEM (45 mM HEPES pH 6.9, 45 mM PIPES pH 6.9, 10 mM EGTA, 5 mM MgCl_2_, 1 mM PMSF) for 1 minute at room temperature. After an additional wash with 1X PBS, the cells were incubated with a conformational monoclonal Active-RAN antibody (26915, NewEastBioscences) diluted 1:50 in PBS/0.1% Tween containing 3% BSA for 2 h at room temperature. The cells were then fixed with 3.7% formaldehyde and 30 mM sucrose for 10 minutes, washed twice with 1X PBS, post-fixed with ice-cold methanol and re-washed twice with 1X PBS. The prepared samples were incubated with TRITC-conjugated goat anti-mouse Ig (A11004, Molecular Probes diluted 1:250) to detect Active-RAN in dilution buffer containing 3% BSA for 30 minutes and, finally, incubated with 4′,6-diamidino-2-phenylindole (0.05% μg/ml) for 1 minute at room temperature to stain DNA. The samples were washed three times with PBS/Tween 0.1% before images acquisition. Samples were visualized under a Leica TC SP2 microscope (Leica, Wetzlar, Germany) with a x63 objective and processed with Leica confocal software. The digital zoom is indicated by the scale bar. Immunofluorescence analyses of RANBP1 and RANGAP1 were performed as follows. HUH7 EGFP cells, HUH7 cells treated with SI113 or DMSO for 24 h and HUH7 cells overexpressing SGK1 were grown on coverslips in 6-well culture dishes in Dulbecco’s Modified Eagle’s Medium (Invitrogen). The cells were subsequently fixed in 3.7% formaldehyde for 20 minutes, permeabilized in 0.5% Triton X-100 for 5 minutes and washed with phosphate-buffered saline (PBS) (pH 7.4). Next, the samples were incubated with a goat polyclonal antibody for total RAN (Santa Cruz sc-1156, 1:250 dilution), a goat RANBP1 antibody (sc-1160, Santa Cruz, 1:250 dilution) and a mouse RANGAP1 antibody (sc-28322, Santa Cruz, 1:250 dilution) for 2 h at room temperature in PBS (pH 7.4) containing bovine serum albumin (1 mg/ml) and Tween-20 (0.2%). The cells were then washed in PBS 1X and incubated with FITC-conjugated donkey anti-goat Ig (sc-2024, Santa Cruz) to detect RAN, TRITC-conjugated donkey anti-goat Ig (705-586-147, Jackson Immunoresearch) to detect RANBP1 and a TRITC-conjugated goat anti-mouse antibody (molecular probes A11004) to detect RANGAP1, in the presence of 4′,6-diamidino-2-phenylindole (0.05% μg/ml). The samples were finally visualized under a Leica TC SP2 microscope (Leica, Wetzlar, Germany) with a x63 objective and processed with Leica confocal software. The digital zoom is indicated by the scale bar.

### Viability assay

Cells (1.5 × 10^4^) were plated in 60-mm tissue culture dishes and allowed to attach for 24 h. Control (EGFP empty vector), pRANBP1-EGFP and pRANGAP1-EGFP HUH7 cells were subsequently incubated in the absence or presence of 12.5 μM SI113 for 72 h. Cell proliferation and viability were assessed using a Countess™ automated cell counter (Catalog no. C10227, Invitrogen) with trypan blue staining.

### Mouse xenograft model

Female NOD/SCID mice (4-week-old, Harlan, Indianapolis, IN) were maintained under pathogen-free conditions and given food/water *ad libitum*. At 6 weeks of age, mice were subcutaneously injected with 2.5 × 10^6^ HUH7 cells, treated and processed as described in ref. 54.

### Statistical analysis

All tests were performed at least in triplicate, and all experiments were performed at least three times. The results are expressed as the mean ± standard deviation (SD). Differences between groups were analyzed using Student’s *t*-test or one-way analysis of variance (ANOVA), followed by Bonferroni’s test for multiple comparisons. The analysis was conducted using GraphPad Prism software (San Diego, CA, USA), and differences were considered significant at *P ≤ 0.05, **P ≤ 0.01, and ***P ≤ 0.001.

### Ethical statement

The experimental protocols have been approved by Research Committee of Department of Human Healt (University “Magna Graecia” of Catanzaro). Animal experiments were carried out in accordance with the Catanzaro University Institutional Animal Care and Use Committee guidelines, using an approved protocol.

## Additional Information

**How to cite this article:** Dattilo, V. *et al*. SGK1 affects RAN/RANBP1/RANGAP1 via SP1 to play a critical role in pre-miRNA nuclear export: a new route of epigenomic regulation. *Sci. Rep.* **7**, 45361; doi: 10.1038/srep45361 (2017).

**Publisher's note:** Springer Nature remains neutral with regard to jurisdictional claims in published maps and institutional affiliations.

## Supplementary Material

Supplementary Information

## Figures and Tables

**Table 1:** Primers list for pre- and pri-miRNAs.

miRNA precursor (pre-miRNAs)	Sense primer (5′ → 3′)	Antisense primer (5′ → 3′)	Tm primers (Sense/Antisense) °C	Amplicon size	Reference primers
hsa-miR-103	TGCCTTCATAGCCCTGTACAA	TTACAGTGCTGCCTTGTTGC	59,09/59,33	58 bp	
hsa-miR-182	TTTGGCAATGGTAGAACTCAC	GTTGGCAAGTCTAGAACCACC	56,44/58,85	63 bp	68
hsa-miR-191	GCAACGGAATCCCAAAAGCA	AGAGCAGGGGACGAAATCCA	59,68/60,91	71 bp	
hsa-miR-223	CCGTGTATTTGACAAGCTGAGT	TGGGGTATTTGACAAACTGACA	58,93/57,83	65 bp	68
SNORD49A	ATCACTAATAGGAAGTGCCGTC	AGACAGGAGTAGTCTTCGTCAG	57,68/58,66	55 bp	
**miRNA precursor (pri-miRNAs)**	**Sense primer (5′ → 3′)**	**Tm primers°C**	**Amplicon size**		
hsa-miR-103	CATTTGGAAGGCAGCTATGCTC	59,97	157 bp		
hsa-miR-182	CCATCCTAACTGTCTCTGTCTC	57,35	146 bp		
hsa-miR-191	CTCTAGACTCCGTTTCACAACC	58,42	205 bp		
hsa-miR-223	GGGTGTGACTTCATCATTCCTT	58,31	175 bp		
SNORD49A	GTCTCCAGTAGCAGGGTTAGC	59,86	153 bp		

**Table 2:** Primers list for the selected genes.

mRNA	Sense primer (5′ → 3′)	Antisense primer (5′ → 3′)	Tm primers (Sense/Antisense) °C	Amplicon size	Reference primers
hsa-RANGAP1	TCAAGAGCTCAGCCTGCTTC	TTCCGGTGACATTCGGTCAG	60.04/60.04	109 bp	
hsa-RANBP1	ATGCGGGCAAAACTGTTCCGAT	ATGGCCCCTTTCTCCTTGTGCT	60.00/60.50	107 bp	
hsa-SGK1	GGCACCCTCACTTACTCCAG	GGCAATCTTCTGAATAAAGTCGTT	59.75/57.82	102 bp	
mm-RANGAP1	GTTTGTGGCTGGCAGAAATCG	TCTGTGGCATATGCACCTCTTC	60.67/60.42	101 bp	
mm-RANBP1	AGATGCGTGCAAAGCTGTTC	GGTTGGCGCATATCTTCAAGG	59.76/59.67	156 bp	
hsa-HPRT	TCGCTTCGGCAGCACATATAC	TGGAACGCTTCACGAATTTGC	60.87/60.33	94 bp	69
mm-HPRT	AGCGTCGTGATTAGCGATGA	CTCGAGCAAGTCTTTCAGTCCT	59.62/60.03	136 bp	
