# Supplementary material for: SGK1 affects RAN/RANBP1/RANGAP1 via SP1 to play a critical role in pre-miRNA nuclear export: a new route of epigenomic regulation
Source: Sci Rep. 2017 Mar 30;7:45361. doi: 10.1038/srep45361 (PMC5371792; doi:10.1038/srep45361)
Supplement: Supplementary Information [file srep45361-s1.pdf]

SGK1 affects RAN/RANBP1/RANGAP1 via SP1 to play a critical role in pre-miRNA nuclear export: a hypothesis for a new route of epigenomic regulation.

Vincenzo Dattilo [1*], Lucia D'antona[1*], Cristina Talarico [1], Mjriam Capula [1], Giada Catalogna[1], Rodolfo Iuliano [2], Silvia Schenone [3], Sante Roperto[4], Cataldo Bianco[2], Nicola Perrotti [1#] and Rosario Amato [1#]

Supplementary file- Figure Legend

Suppl. File 1 qRT-PCR evaluation for exogenous SGK1, RANBP1, RANGAP1 expression, and SGK1 and RANBP1 silencing.Graphs represent the fold increase values +/- SD of the either transduced/transfected or silenced genes, except for SGK1 silencing which values are presented as percentage of fold expression +/- SD. Statistical significance has been calculated as detailed in the Methods section. *$P \leq 0.05$; **$P \leq 0.01$; ***$P \leq 0.001$.

Suppl. File 2 Western blot analysis of HUH7 stably overexpressing EGFP-SGK1 or EGFP in presence or not of transiently silencing for SP1 (shSP1); western blot of RANBP1-silcenced or scrambled HUH7 with or without 12.5 μM SI113 treatment for 72 h; western blot of HUH7 cells stably silenced SGK1 or scrambled.Cell extracts were loaded on SDS-polyacrylamide gel electrophoresis for immunoblotting using SP1 antibody, RANBP1 antibody, SGK1 antibody and GAPDH antibody.

Suppl. File 3 Quantitative RT-PCR analysis of mature microRNAs in the cytoplasm of HUH7 cells stably over-expressing EGFP-SGK1 or EGFP, with or without transiently silenced RANBP1 (shRANBP1).Statistical significance has been calculated as detailed in the Methods section. *$P \leq 0.05$; **$P \leq 0.01$; ***$P \leq 0.001$.

Suppl. File 4 Cell viability analysis by The Countess™ automated cell counter in HUH7-GFP and HUH7-RANBP1-GFP (top panel) and HUH7-GFP,HUH7-RANBP1-GFP, HUH7-RANGAP1 and HUH7-RANBP1/RANGAP1 (bottom panel) cell lines 72 h after treatment with either SI113 at the indicated concentrations or vehicle alone. Results are reported as means of three independent experiments, each conducted in triplicate, and expressed as the percentage of viable control cells treated with DMSO alone (vehicle). The fluorescence microscopy for RANBP1-GFP expression is shown at the top of the panel.

Suppl. File 5 Representative immunofluorescence analysis of HUH-EGFP cells (top panel), no differences was recordable with the scrambled control cells, reported in Fig 6d and e. Samples were incubated with a conformational Active-RAN monoclonal antibody, or with an anti-RANBP1 goat polyclonal antibody or an anti-RANGAP1 monoclonal antibody and 4',6-diamidino-2-phenylindole (0.05% µg/ml). Histograms representing the quantification of immunofluorescence showed in Fig. 6d and 6e (bottom panel). The values are plotted as relative IF unit expressed as percentage of RAN-GTP/RAN total ratio (left), RANBP1 (middle) and RANGAP1 (right).

Suppl. File 1

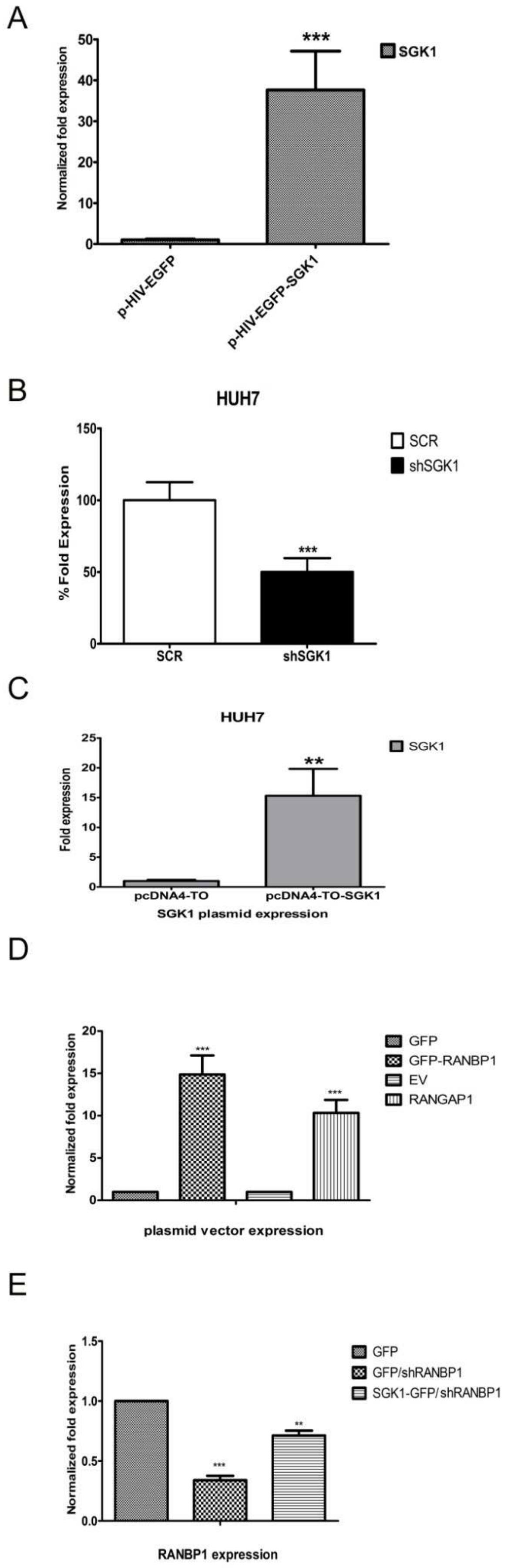
A
Normalized fold expression
50
40
30
20
10
0
***
SGK1
p-HIV-EGFP
p-HIV-EGFP-SGK1
B
HUH7
% Fold Expression
150
100
50
0
***
SCR
shSGK1
SCR
shSGK1
C
HUH7
Fold expression
25
20
15
10
5
0
**
SGK1
pcDNA4-TO
pcDNA4-TO-SGK1
SGK1 plasmid expression
D
Normalized fold expression
20
15
10
5
0
***
***
GFP
GFP-RANBP1
EV
RANGAP1
plasmid vector expression
E
Normalized fold expression
1.5
1.0
0.5
0.0
***
**
GFP
GFP/shRANBP1
SGK1-GFP/shRANBP1
RANBP1 expression

Suppl. File 2

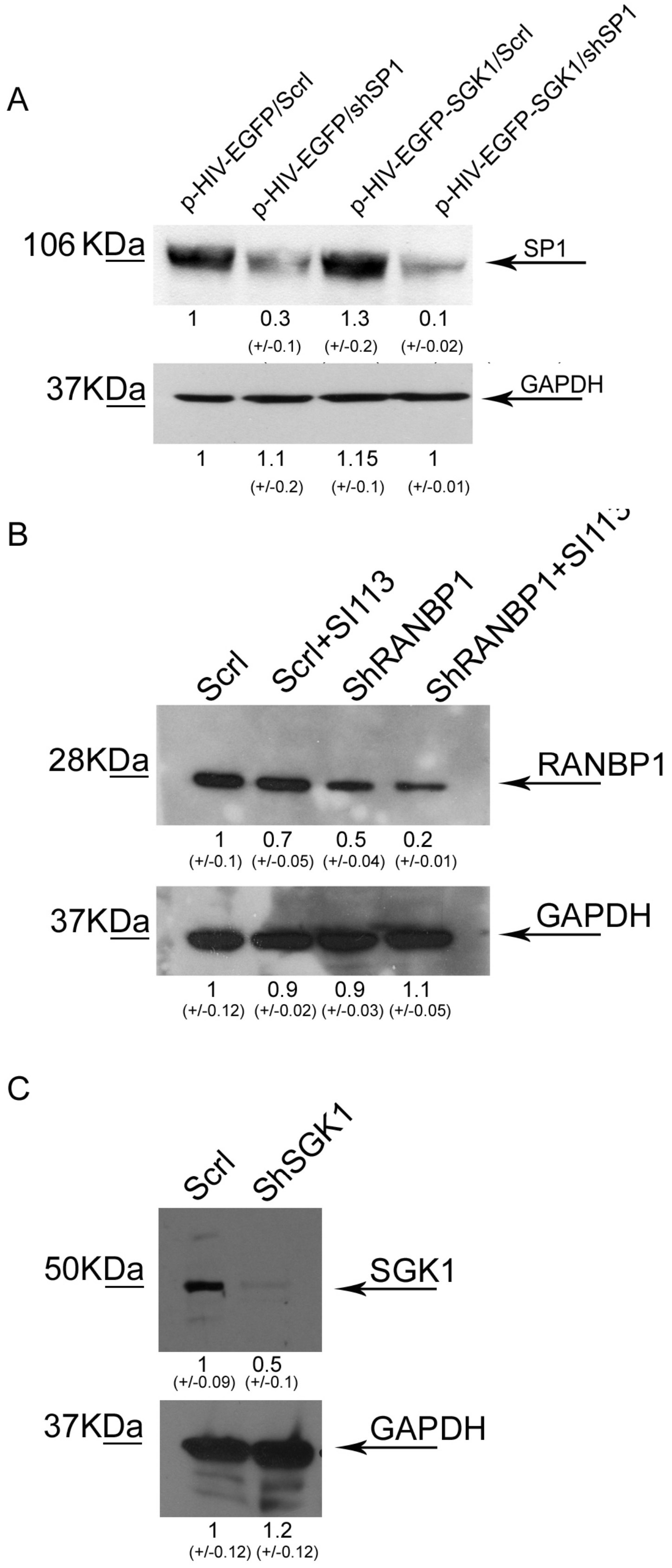
A
p-HIV-EGFP/Scrl
p-HIV-EGFP/shSP1
p-HIV-EGFP-SGK1/Scrl
p-HIV-EGFP-SGK1/shSP1
106 KDa
SP1
1
0.3 (+/-0.1)
1.3 (+/-0.2)
0.1 (+/-0.02)
37KDa
GAPDH
1
1.1 (+/-0.2)
1.15 (+/-0.1)
1 (+/-0.01)
B
Scrl
Scrl+SI113
ShRANBP1
28KDa
RANBP1
1 (+/-0.1)
0.7 (+/-0.05)
0.5 (+/-0.04)
0.2 (+/-0.01)
37KDa
GAPDH
1 (+/-0.12)
0.9 (+/-0.02)
0.9 (+/-0.03)
1.1 (+/-0.05)
C
Scrl
ShSGK1
50KDa
SGK1
1 (+/-0.09)
0.5 (+/-0.1)
37KDa
GAPDH
1 (+/-0.12)
1.2 (+/-0.12)

Suppl. File 3

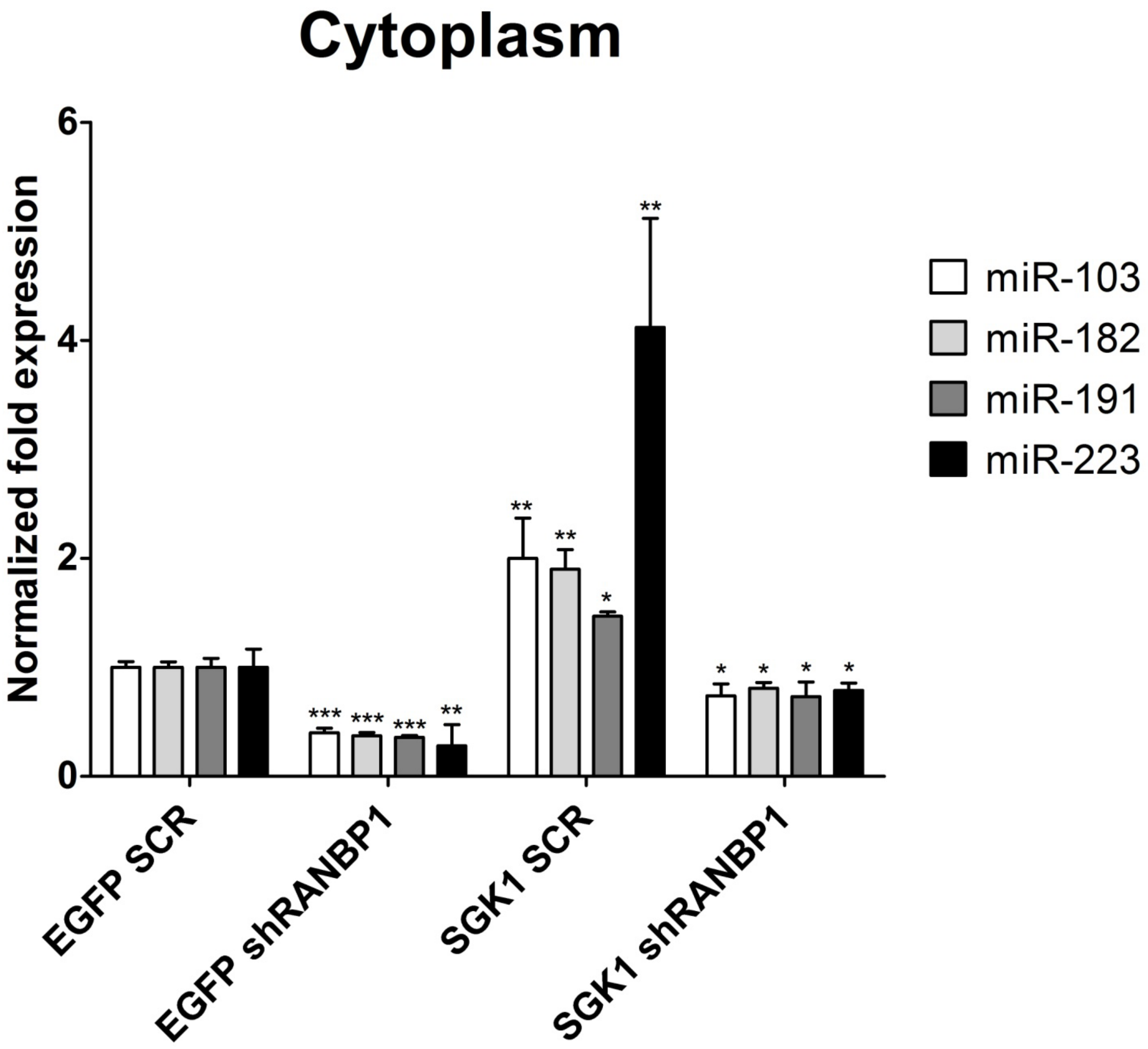
Cytoplasm
Normalized fold expression
0
2
4
6
**
**
**
*
***
***
***
**
*
*
*
*
miR-103
miR-182
miR-191
miR-223
EGFP SCR
EGFP shRANBP1
SGK1 SCR
SGK1 shRANBP1

Suppl. File 4

A

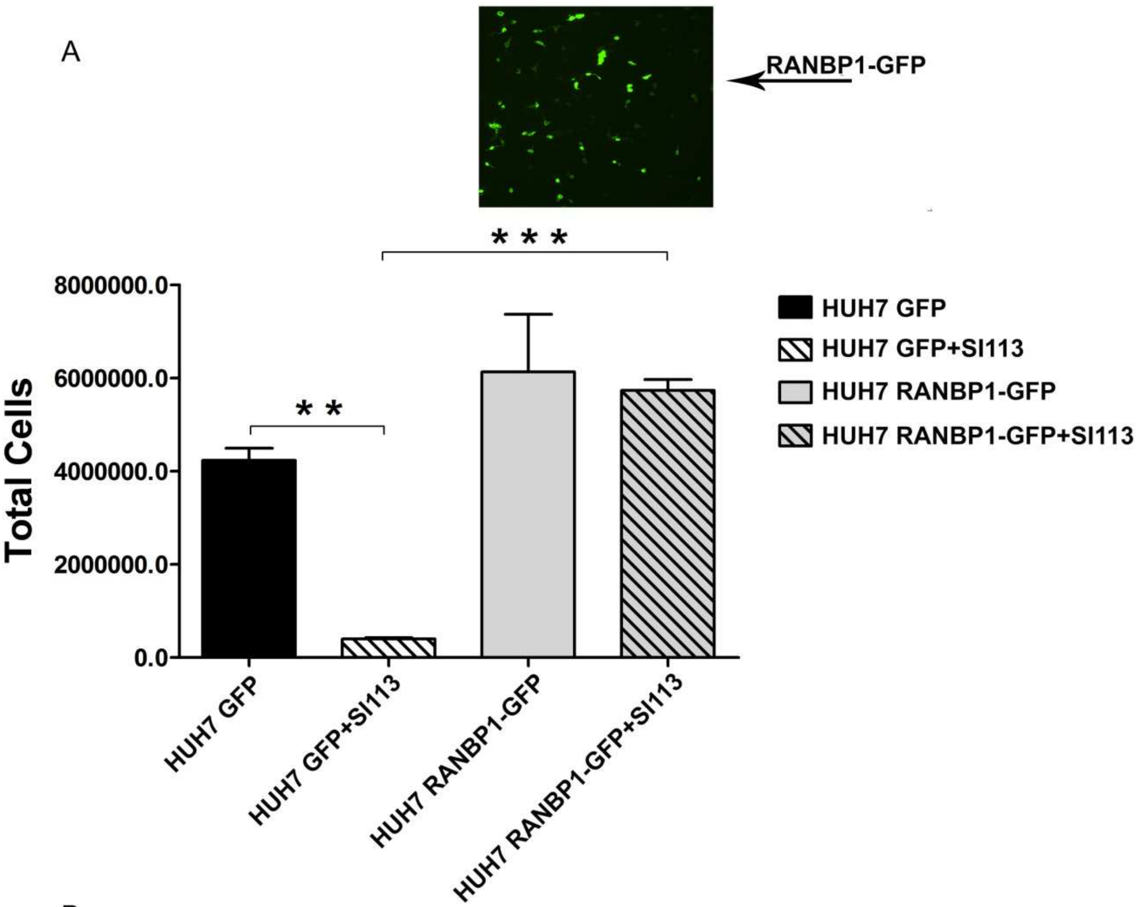

B

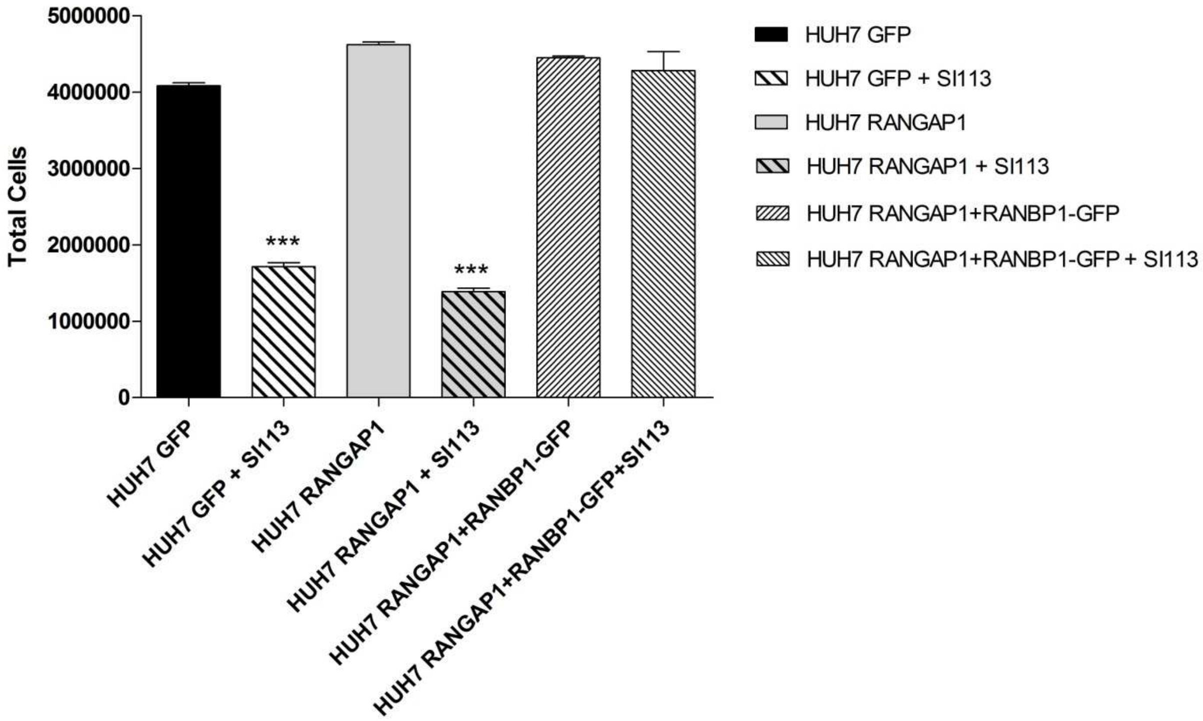

Suppl. File 5

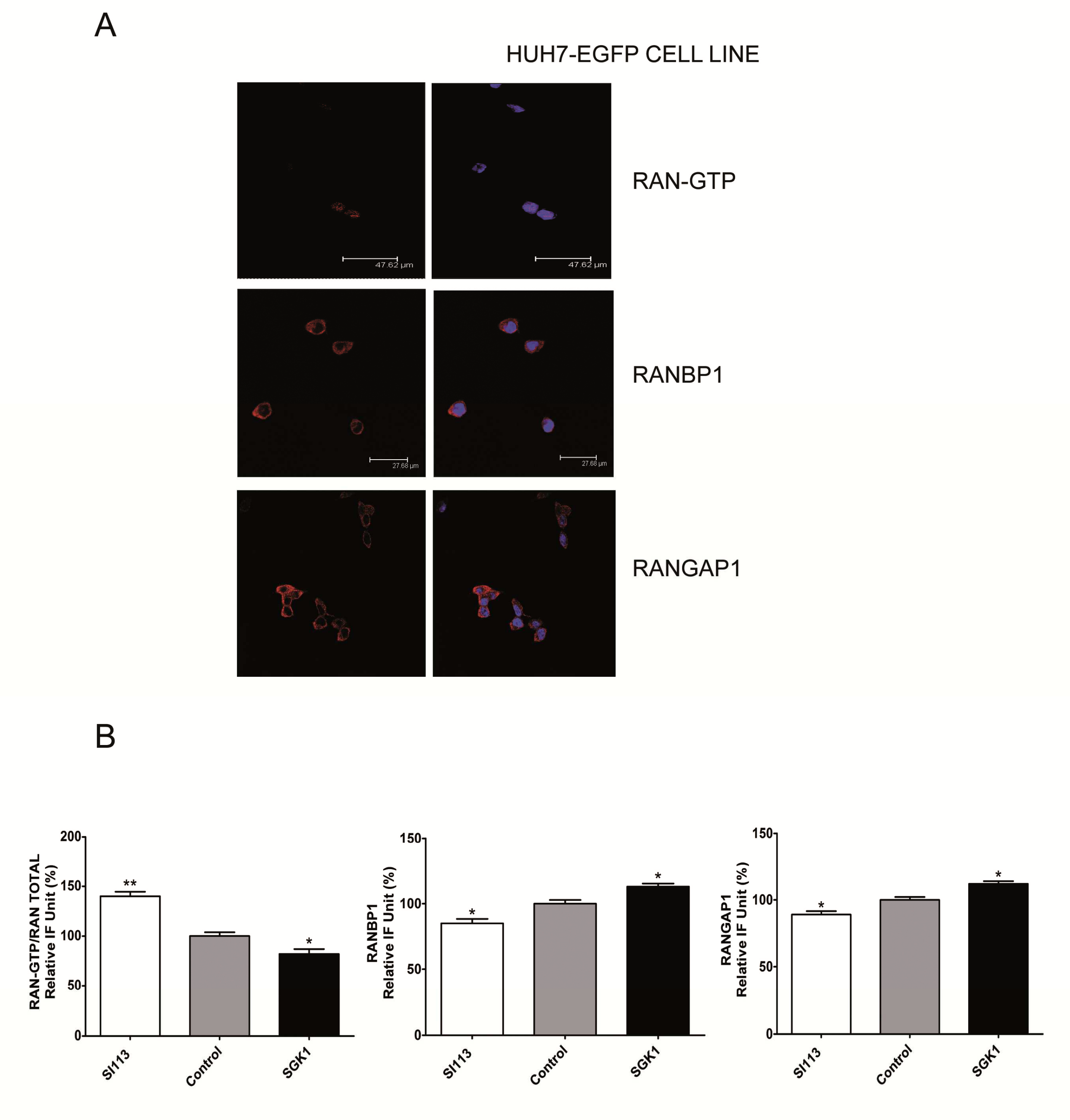
A
HUH7-EGFP CELL LINE
RAN-GTP
RANBP1
RANGAP1
47.62 µm
27.68 µm
B
RAN-GTP/RAN TOTAL Relative IF Unit (%)
RANBP1 Relative IF Unit (%)
RANGAP1 Relative IF Unit (%)
0
50
100
150
200
**
*
SI113
Control
SGK1

Raw gels Figure 5

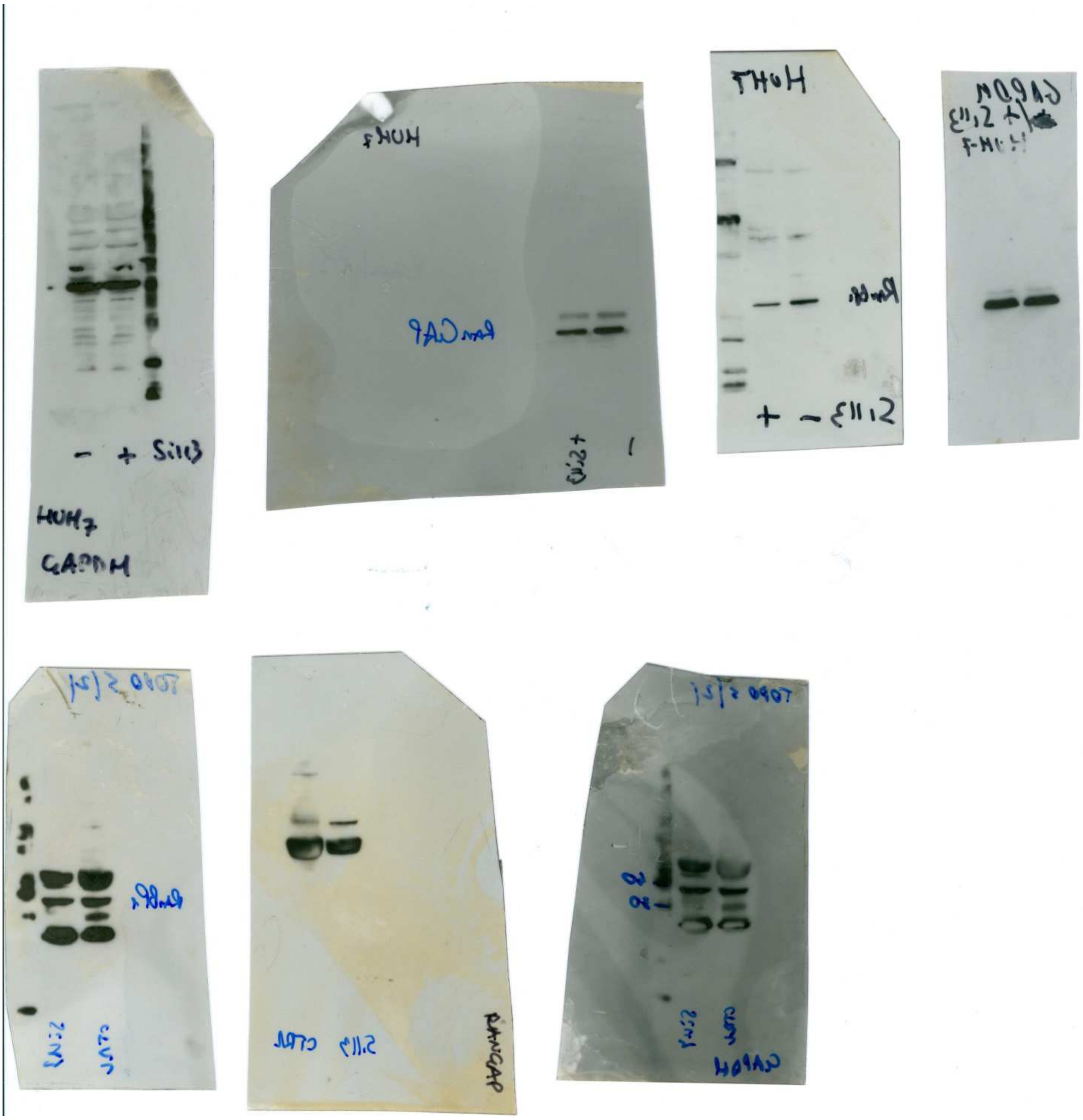

Raw gels Figure 6

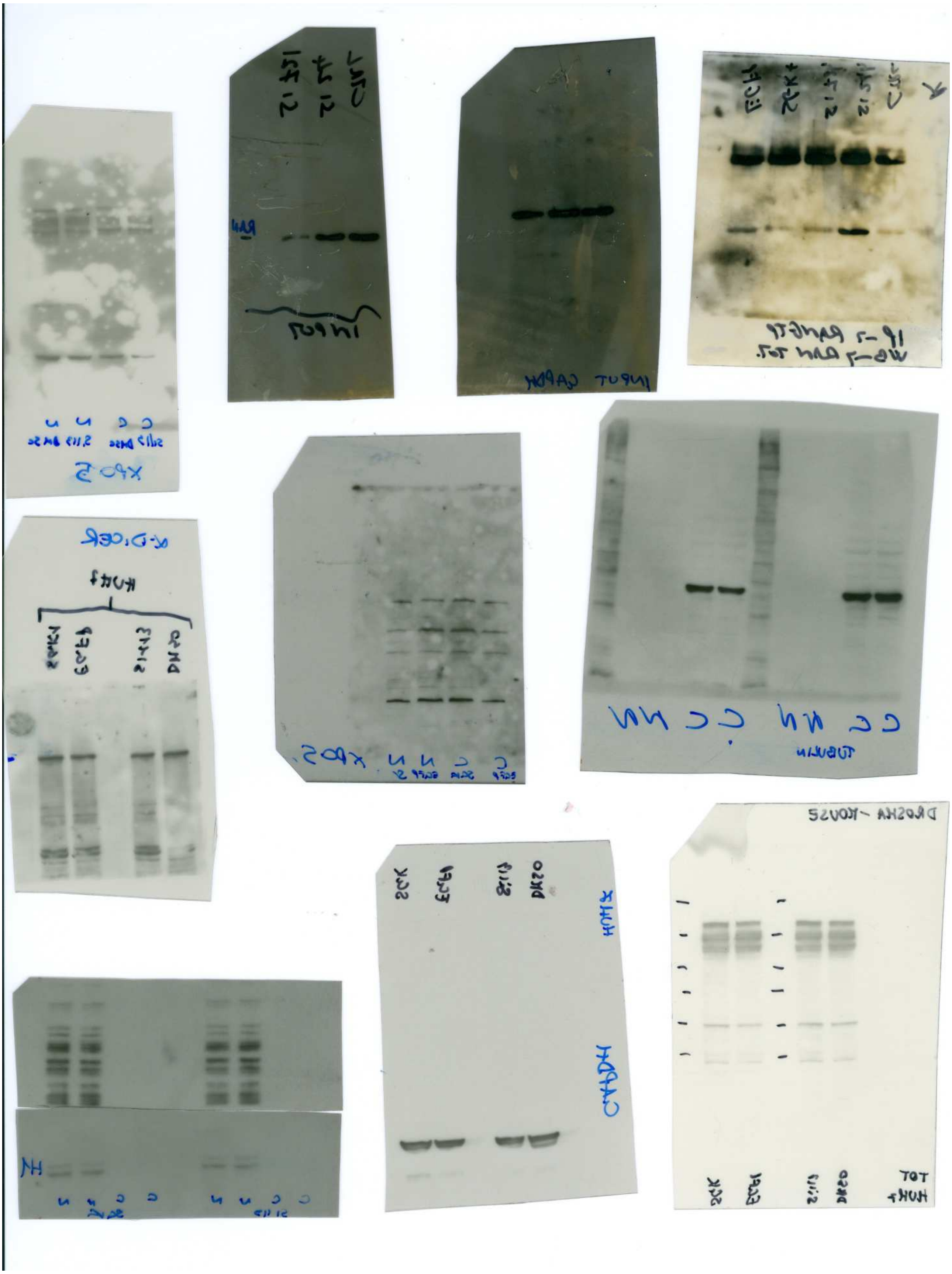

Raw gels SF 2

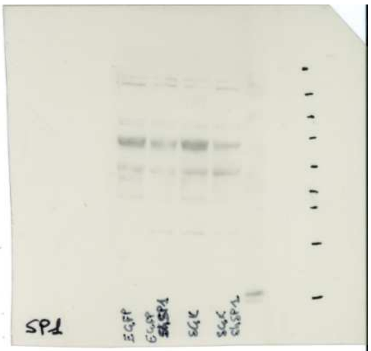
SP1

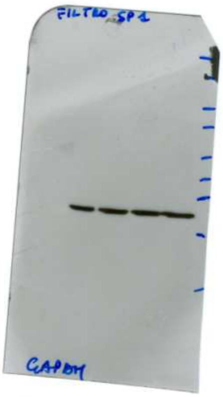
FILTRO SP1
GAPDH

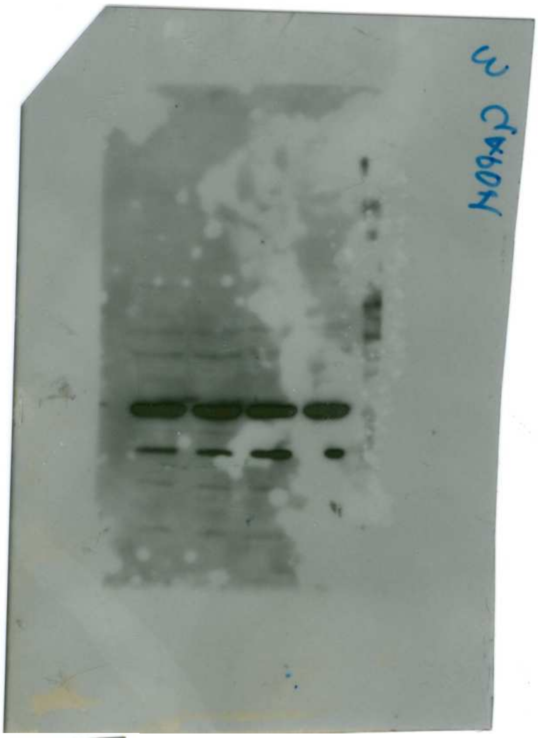

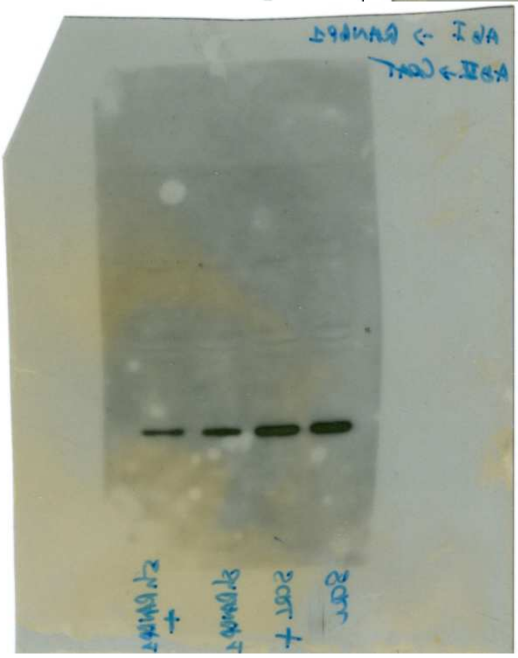

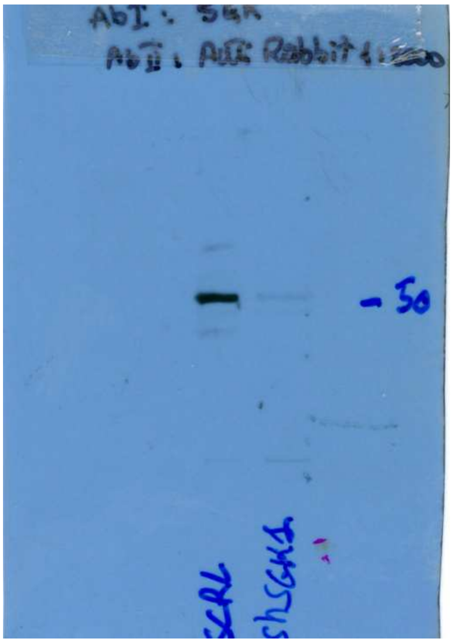
50
SCRL
shSGK1

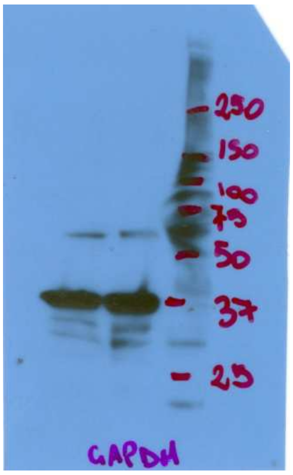
250
150
100
75
50
37
25
GAPDH
